# The Epidemiological Impact of Dengue in Colombia: A Systematic Review

**DOI:** 10.4269/ajtmh.23-0907

**Published:** 2024-10-29

**Authors:** Alfonso J. Rodríguez-Morales, Eduardo López-Medina, Iván Arboleda, Jaime A. Cardona-Ospina, Jaime E. Castellanos, Álvaro A. Faccini-Martínez, Elaine Gallagher, Riona Hanley, Pio Lopez, Salim Mattar, Carlos Eduardo Pérez, Randee Kastner, Humberto Reynales, Fernando Rosso, Jing Shen, Wilmer E. Villamil-Gómez, Marcela Fuquen

**Affiliations:** ^1^ Grupo de Investigación Biomedicina, Faculty of Medicine, Fundación Universitaria Autónoma de las Américas-Institución Universitaria Visión de las Américas, Pereira, Colombia;; ^2^ Master of Clinical Epidemiology and Biostatistics, Universidad Científica del Sur, Lima, Perú;; ^3^ Colombian Association of Infectious Diseases (ACIN), Bogotá, Colombia;; ^4^ Department of Pediatrics, Universidad del Valle, Cali, Colombia;; ^5^ Centro de Estudios en Infectología Pediátrica, Cali, Colombia;; ^6^ Clínica Imbanaco, Grupo Quirón Salud, Cali, Colombia;; ^7^ Baxalta Colombia SAS, (Takeda), Bogotá, Colombia;; ^8^ Grupo de Investigación en Infecciones Emergentes y Medicina Tropical, Instituto para la Investigación en Ciencias Biomédicas, SCI-HELP, Pereira, Colombia;; ^9^ Universidad El Bosque, Vicerrectoría de Investigación, Grupo de Virología, Bogotá, Colombia;; ^10^ Servicio de Infectología, Hospital Militar Central, Bogotá, Colombia;; ^11^ Facultad de Medicina, Universidad Militar Nueva Granada, Bogotá, Colombia;; ^12^ Takeda Pharmaceuticals International AG, Zurich, Switzerland;; ^13^ Instituto de Investigaciones Biológicas del Trópico, Universidad de Córdoba, Montería, Colombia;; ^14^ Servicios y Asesorías en Infectología, Bogotá, Colombia;; ^15^ Centro de Atención e Investigación Médica – CAIMED, Chía, Colombia;; ^16^ Fundación Valle del Lili, Infectious Diseases Service, Cali, Colombia;; ^17^ Universidad Icesi - Facultad de Ciencias de la Salud, Cali, Colombia;; ^18^ Universidad Simón Bolívar, Centro de Investigación en Ciencias de la Vida, Barranquilla, Colombia

## Abstract

Dengue is the most important viral vector-borne disease in the tropics, with Colombia being one of the most affected countries. In this context, it is essential to identify and synthesize the existing evidence on the epidemiology of dengue in Colombia. A systematic review (PROSPERO CRD42021257985) was conducted by searching for epidemiological data in populations with suspected or confirmed dengue in Colombia from 2012 to 2020. We searched PubMed, EMBASE, the Cochrane Library, the LILACS, and SciELO databases, and 104 publications out of 1,234 records were selected. The dengue annual incidence rate varied through the years without a clear trend. The lowest annual incidence rate was observed in 2017 (90.7 per 100,000 population) and the highest in 2013 (476.2 per 100,000 population). The proportion of severe cases in the same period ranged between 0.89% in 2016 and 2.7% in 2012. The four dengue virus (DENV) serotypes co-circulated in the country, and DENV-2 was the predominant serotype. Fifty percent of dengue cases occurred in people under 20 years, and those between 5 and 14 years had the highest incidence rate. The mortality rate for all dengue cases ranged from 0.07% in 2020 to 0.16% in 2012 and 2015. In conclusion, dengue is a hyperendemic disease in Colombia with the circulation of four serotypes. New strategies must be implemented to prevent the contagion and impact of the disease on the population at risk.

## INTRODUCTION

Dengue is a viral infection transmitted to humans through the bite of *Aedes aegypti* and *Aedes albopictus* mosquitoes. Dengue is present in at least 129 countries worldwide, particularly in urban and semi-urban areas in tropical and subtropical climates. Its worldwide incidence has grown dramatically in recent decades, with about 390 million children and adults infected annually WHO. Of those infected, a quarter develop clinical manifestations with a spectrum of symptoms of varying severity. Every year, around 500,000 people with severe dengue (SD) require hospitalization, and an estimated 2.5% of these die.^1^

Multiple dengue infections are possible owing to the four existing serotypes: dengue virus (DENV)-1, DENV-2, DENV-3, and DENV-4. All could produce overt illness, with fever, headache, eye pain, muscle aches, nausea, vomiting, and rash lasting days to weeks. Symptomatic infected persons often seek care from healthcare personnel who monitor the evolution and development of complications or organ dysfunction. In some cases, supportive hospital care is needed.^2^ Early detection of disease progression and access to appropriate medical care can reduce mortality rates of SD to less than 1%.

**Figure 1.**
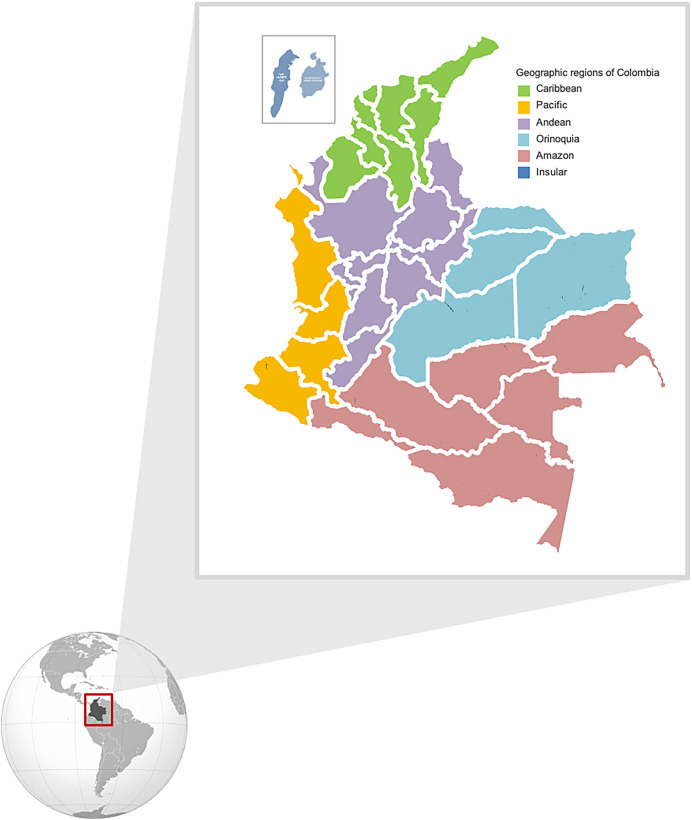
Map of Colombia. Colombia is a country that is divided into six regions: Caribbean, Andean, Orinoquia, Amazon, Pacific coast, and Insular. Each region has “departments” (*N* = 32) (a political and administrative division of the country). Each department is divided into “municipalities” (*N* = 1.103).

The incidence of dengue, specifically in the Americas, has continued to increase, and Colombia, located in a tropical zone, is one of the most affected countries, with four circulating dengue virus serotypes and epidemics every 3–4 years.^3^ The Andes Mountains cross Colombia, and it has coasts on both the Atlantic and Pacific oceans. Because it is very close to the equator at a low latitude, all of Colombia’s climates are isothermal, with no significant thermal amplitude throughout the year (a difference of less than 5°C between the coldest and warmest months), so there are no seasons. However, there are seasons according to precipitation (dry and rainy seasons). In Colombia, altitude (meters above sea level) determines climate. The warm zone is below 1,000 meters (3,281 feet) of altitude, with temperatures above 24°C (75.2°F). About 82.5% of the country’s area lies in the warm altitudinal zone. The zone between 1,001 and 2,000 meters (3,284 and 6,562 feet) presents an average temperature between 17°C and 24°C (62.6°F and 75.2°F). The cold climate is between 2,001 and 3,000 meters (6,565 and 9,843 feet), and the temperatures vary between 12°C and 17°C (53.6°F and 62.6°F). Thermal floors are relevant owing to the distribution of the vector, as *Ae. aegypti* predominates in areas closer to sea level (Figure 1). Colombia’s distinctive characteristics in terms of latitude, altitude, temperature, and precipitation make the country a hyperendemic place for DF. In addition, although dengue infection is notifiable in the country, there is an underreporting of cases that limits knowledge of the actual situation of the disease and its burden.^4^

Prevention strategies for dengue are of great importance. Still, currently, they are mainly based on vector control measures (prevention of breeding and use of personal protection against bites), which have proven to be generally ineffective.^1^ The WHO in 2020 included dengue in a list of urgent health challenges for the next decade after it had been considered one of the top 10 threats to public health worldwide. Dengue infection is associated with a significant disease burden for patients, caregivers, and society. Therefore, a systematic literature review (SLR) was conducted to identify and synthesize the existing evidence on the burden of dengue in Colombia.

## MATERIALS AND METHODS

### Search strategy, eligibility criteria, and study selection.

This systematic review was registered to PROSPERO (CRD42021257985), and it followed the Cochrane Handbook for Systematic Reviews and the Preferred Reporting Items for Systematic Reviews and Meta-Analyses (PRISMA). In April 2021, we completed a comprehensive search for dengue epidemiology in Colombia using PubMed, EMBASE, the Cochrane Library, Latin American and Caribbean Health Sciences Database (LILACS), and Scientific Electronic Library Online (SciELO). As a search algorithm, we used (“dengue” OR “severe dengue” OR “dengue virus” OR “dengue hemorrhagic fever”) combined with terms related to epidemiology and disease burden (see Supplemental Material). We restricted our search to articles in English or Spanish published from 2012 to 2020, given that an SLR of the epidemiology of dengue in Colombia was conducted from 2000 to 2011.^4^ We conducted a targeted search in grey literature on the websites of government and public health agencies, the leading universities in Colombia, and conferences to supplement the electronic database searches and reduce publication bias (see Supplemental Appendix).^5^

We included studies of any design in individuals of all ages with suspected or confirmed dengue in Colombia, which reported epidemiological data. We excluded publications that did not clearly outline methods and sources for data collection or analysis, including news and opinion articles, case reports, narrative reviews, and letters.

Two investigators independently screened all the references retrieved by title and abstract. Discrepancies were discussed, and a third reviewer made the final decision if not resolved. All citations found during the searches were stored in a reference database. In Microsoft Excel, epidemiology data were collected in separate data extraction forms.

### Quality assessment.

We assessed the methodological quality of peer-reviewed publications only. Epidemiological studies were evaluated using the NIH tool.

### Data extraction and synthesis of results.

We performed a descriptive summary of the extracted data, prioritizing annual data obtained from the Colombian National Institutes of Health (INS – Spanish for Instituto Nacional de Salud) database. Where not available, monthly data and data from other surveillance sources and peer-reviewed journals were used. The most recent publication was considered for studies based on the same cohort and similar results.

## RESULTS

A total of 1,234 records were identified from electronic databases and 267 from the grey literature searches. Of these, 104 publications were selected for data extraction and inclusion in this section (PRISMA diagram in Supplemental Figure 1 in the Supplemental Material). The characteristics of the included studies are available in the Supplemental Material.

Overall, the quality of 75 studies was assessed using the NIH quality assessment checklist for observational cohort and cross-sectional studies. Twenty-one studies had poor quality, 44 had acceptable quality, and 10 had good quality. The primary reasons for poor scoring were that many studies did not perform statistical analyses, independent variables were not clearly defined, outcome measures were not adequately measured, and confounding variables were not adjusted. The results are provided in the Supplemental Appendix.

### National and regional incidence.

**Figure 2.**
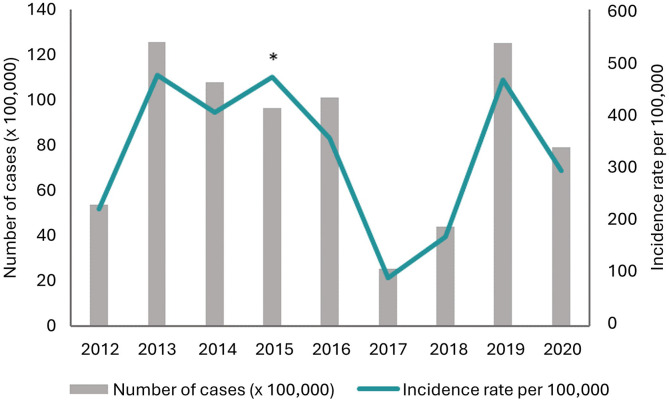
National incidence and number of dengue cases in Colombia 2012–2020. Modified from Colombian NIH data; ReliefWeb. *Incidence rate for 2015 (weeks 1–50) was obtained from ReliefWeb.

In Colombia, all cases of dengue (clinically suspected, laboratory-confirmed, or probable) should be notified to the National Epidemiological Surveillance Information System of the INS (see the Supplemental Appendix for more information). The data summarized in this section are from this source. Overall, the review showed that dengue is hyperendemic in Colombia. From 2012 to 2020, the annual incidence rate of all suspected cases of dengue ranged from 90.7 to 476.2 per 100,000 population, with the most recent outbreak reported in 2019 (465.9 per 100,000 population), corresponding to a sharp increase from the previous 2 years. Main outbreaks were recorded in 2013, 2016, and 2019 (Figure 2). The peak incidence between 2012 and 2020 was reported in 2013 (476.2 per 100,000 population). The lowest incidence rates were reported in 2017 (up to 284.3 cases per 100,000 population). Throughout the review period, the number of reported dengue cases varied from 25,284 to 125,554 and were similar across the different data sources, except for the year 2018 (18,037 in WHO; 44,825 in Pan American Health Organization (PAHO); 44,171 in the INS). The INS did not report nationwide incidence data for 2015; therefore, the data from Relief Web were used (incidence rate, 471.3 per 100,000 population, 96,444 cases).

Dengue is endemic in most regions of Colombia, and the central and southern areas of the country report higher incidence rates. At a territorial level, the annual incidence rates of dengue for 2012–2020 ranged from 0 to 5,881.1 cases per 100,000 population, being higher in regions such as the Amazonas (5,881.1 per 100,000 population), Orinoquia (2,156–1,960 per 100,000 population), and in some departments of the Andean region (1,534.9–1,514.4 per 100,000 population). However, in areas above 2,200 meters above sea level (m.a.s.l.), where the capital city Bogota is, there is no transmission of dengue because there are no vectors at these altitudes, thus explaining the registered incidence rates of 0 per 100,000 population.

There is no significant seasonality in Colombia as dengue cases were observed throughout the year, and the epidemic period varied from year to year.

### Severity.

Dengue severity was classified according to the 2009 WHO dengue classification as dengue fever (DF), dengue hemorrhagic fever (DHF), dengue shock syndrome, dengue with warning signs (DWS), dengue without warning signs (DwWS), and SD. At a national level, from 2012 to 2020, 97.5% of the cases corresponded to DF, and the remaining cases to SD. The proportion of SD cases was similar across the different data sources, ranging from 0.89% to 2.7%.^5^^,^^6^ From 2015 onward, the INS reported DF as DWS and DwWS separately. Of the total cases, approximately 55% corresponded to DwWS and 45% to DWS.

Regarding regional incidence, according to INS data, most of the cases of DF were reported in the southern region of the country, whereas the northern and some of the eastern areas of Colombia reported very low rates of DF cases. A trend similar to the DF cases was observed for SD cases in regions reporting the highest and lowest proportion of cases. For DWS, the regions reporting higher proportions of DWS were also the same as for DF and SD cases.

Disease severity based on comorbidities, morbidity, or disability rates was not reported in the grey literature nor in any studies included in this review.

### Age distribution.

The Colombian INS reported the incidence of dengue by age group from 2012 to 2019. The data showed that approximately 50% of dengue cases were reported in the population under 20 years of age, with 5- to 14-year-olds being the age group with the highest incidence rate. This distribution was stable across the review period. The INS also reported the age distribution of dengue cases according to severity for different years. For example, in the epidemic year 2013, 9.5%, 15.6%, 15.8%, and 12% of all DF cases were in the age groups of 1–4 years, 5–9 years, 10–14 years, and 15–19 years, respectively. Similar trends were also seen in SD, DwWS, and DWS cases. For the adult population, the age group of 19–24 years had the highest number of reported DF and SD cases.

The total annual regional incidence of dengue by age group was unavailable in the included sources. However, the age stratification of dengue cases at the province/city level was reported in 12 publications. The data showed that dengue was more predominant in the first 20 years of life and less frequent in people over 60 years of age.^6^^–^^10^

Regarding severity, one study from a city in Colombia (Cali) reported proportions of cases for various age groups. For DwWS, the 15–34-year-olds accounted for 41.40% of all cases. Meanwhile, for SD and DWS, the 0–14-year-olds accounted for the maximum proportion of cases (DWS, 35.76% and SD, 37.34%). In the population of 65-year-olds and above, the proportion of patients with SD (17.72%) was higher than those with DwWS (6.20%) and DWS (8.81%).^11^ Sarti et al.^12^ reported DHF incidence in 9–16-year-olds in 2012, 2013, and 2014 to be 1.96, 4.83, and 3.01 per 100,000 population.

### Hospitalization.

The INS reported nationwide hospitalization cases according to dengue case definition. However, it was not consistently reported for each dengue case definition except for DWS. Throughout the period for which data are available, the highest number of DWS hospitalizations was in 2019 (50,887 cases). In 2019, the hospitalization rates were 96.7% for SD, 80.1% for DWS, and 14.9% for DwWS. Few studies reported hospitalization due to dengue, and the rates aligned with those from the INS. Between 2014 and 2018, Hernández and Julian^13^ reported an average yearly dengue hospitalization rate of 48.2%. In 2017, Rico-Mendoza et al.^14^ reported a hospitalization rate of 64% for DWS, whereas in 2020, Cardona-Ospina et al.^15^ reported a hospitalization rate of 76.9% for January to May 2020. Overall, high rates of hospitalization for SD and DWS cases were reported in most regions.

### Mortality and case fatality rates.

The INS and PAHO reported nationwide deaths from dengue. The fatality rate for all dengue cases ranged between 0.16% in 2012 and 2015 to 0.07% in 2020. The INS reported the fatality rate for SD for 2012, 2013, 2014, and 2019 as 6.2%, 5.8%, 6.3%, and 9%, respectively. During 2012, 2013, and 2014, a higher proportion of deaths occurred in those under 14 years of age. From 2015 onward, the highest proportion of deaths occurred in those over 65 years of age (23.23% in 2015, 27.27% in 2017, and 16.4% in 2018). According to PAHO, the case fatality rates (CFRs) of dengue ranged between 0.051% and 0.161% from 2012 to 2020. The highest rates were reported in 2015 (0.161%), followed by 2013 (0.123%). It is important to note that the number of deaths reported varied by source. Some studies reported different nationwide dengue mortality rates and CFRs. In 2013, Stanaway et al.^16^ estimated a mortality rate of 0.26 per 100,000. The age-standardized mortality rate ranged from 0.3 to 0.49 per million population. In the same year, Shepard et al.^17^ estimated a 0.03% fatality was higher in adults than in children. Castrillón et al.^18^ reported a fatality rate of 0.35% for 2012 and 0.12% for 2013. Hernández and Julian^13^ reported a fatality rate of 0.29% for the period from 2014 to 2018, whereas Cardona-Ospina et al.^15^ reported a fatality rate of 0.05% for DF in 2020.

### Seroprevalence.

A total of 21 studies in different populations reported data on dengue seroprevalence based on the detection of serum IgG or IgM. Overall, 14 studies provided seroprevalence data for subjects of all ages. The prevalence of IgM antibodies ranged from 12% to 88%,^19^^,^^20^ whereas the prevalence of IgG antibodies ranged from 0.05% to 100%,^10^^,^^21^^–^^24^ although the testing methods and populations varied across studies, making comparisons difficult to assess.

One study assessed the seroprevalence of dengue in healthy children and adults in urban and rural areas of seven endemic regions in Colombia based on 1,318 processed samples between 2013 and 2015. A prevalence of positive dengue IgG antibodies was reported at 91% in 2013 and 2014 and 82% in 2015.^25^ Other studies reported seroprevalence of IgG antibodies in other regions ranging between 48.9% and 98.2%.^22^^,^^23^

According to a study in seven different endemic regions of Colombia between 2013 and 2015, the age-specific prevalence of IgG antibodies was reported for ages 4–11 years, 12–25 years, 26–45 years, and 46–95 years as 85%, 88.3%, 91.4%, and 94.4%, respectively.^25^ Three studies in Colombian regions with different conditions of temperature, altitude, and population density reported seroprevalence in children only,^20^^,^^21^^,^^24^ 29.1% among suspected dengue cases aged 4–14 years,^24^ 64.6% among confirmed dengue cases in 5–19-year-olds,^21^ and 77.2–88% among suspected dengue cases in <18-year-olds in other Colombian regions.^20^

### Serotype distribution.

Based on PAHO, DENV-1, 2, and 3 circulated in Colombia from 2017 to 2019, whereas from 2012 to 2016 and 2020, all four DENV serotypes were co-circulated in the country.

**Figure 3.**
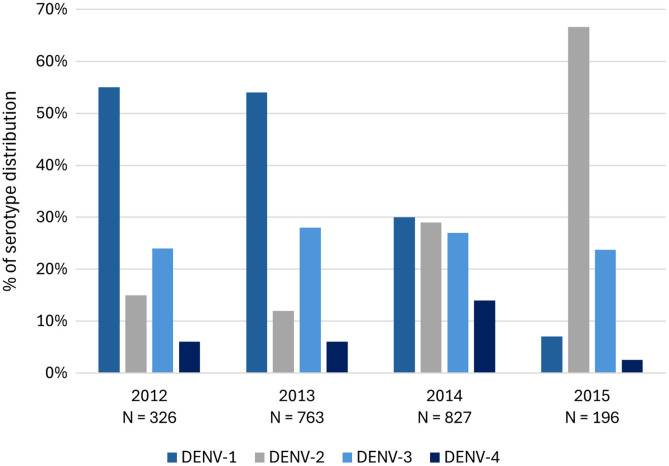
National dengue serotype distribution, 2012–2015.

According to the Colombian INS data, from 2012 to 2014, DENV-1 was the predominant serotype (55%, 54%, and 30% for 2012, 2013, and 2014, respectively), but in 2015, the dominant serotype was DENV-2 (67%). Throughout 2012–2015, DENV-4 was the least dominant (Figure 3). See the Supplemental Appendix for more information.

Information about dengue serotype distribution within different regions and cities in Colombia was available in 16 studies. Overall, the data were consistent with those from the INS and PAHO, with DENV-1, DENV-2, and DENV-3 being the predominant serotypes. Studies conducted in different geographic regions reported variable prevalence of each serotype, DENV-1 from 3.9% to 42.8%, DENV-2 from 4% to 33.3%, DENV-3 from 0% to 70%, and DENV-4 from 0% to 7.9%.^25^^–^^27^

Three studies reported serotype distribution by age. Avilés-Vergara et al.^6^ described the distribution within seven age groups, ranging from <10 to >60 years, in a northern department (Córdoba). For the study period from June 2015 to December 2016, DENV-2 was consistently the most predominant serotype reported in each age group and DENV-4 the least predominant. Dengue virus–2 was significantly higher in children <10 years (40%) compared with 11 to >60-year-olds (2–12%).^6^

### Expansion factors.

Expansion factors are multiplication factors used to correct the underreporting of dengue cases in the passive surveillance system. Of the identified publications, only two reported the expansion factors and underreporting of dengue cases in Colombia. The studies represented four cities’ expansion factors ranging from 2.03 to 5.9. The expansion factors also varied by age, setting, and healthcare provider.^28^

## DISCUSSION

This review presents a compilation of evidence regarding the epidemiological burden of dengue in Colombia. In general, dengue is hyperendemic in the country, with all four serotypes circulating. Main outbreaks were recorded in 2013, 2016, and 2019. Records showed a significant increase in the incidence of dengue. The most recent outbreak recorded in 2019 (257.87 per 100,000 population) represented a substantial increase over the previous 2 years. This same trend occurred in countries such as Brazil, Mexico, Nicaragua, Honduras, and El Salvador, corresponding to the highest number of cases ever recorded for the Americas, which exceeded by 13% the number of cases reported in the 2015 epidemic year, with a higher proportion of SD (0.8%) than the previous 4 years.^29^

In Colombia between 2012 and 2020, more than 750,000 cases of dengue were reported, the annual incidence rate of all suspected dengue cases ranged from 51.53 to 271.90 per 100,000 population, and dengue-related deaths were reported every year. This period corresponded to the Ten-Year Public Health Plan (2012–2021) of the Ministry of Health and Social Protection, which defined as a priority the control of communicable diseases in Colombia and established a goal to reduce or maintain the CFR for SD to <2% by 2021 in the country, departments, and municipalities. This goal was not achieved in many territories where mortality rates in patients with SD remained high. In the country, the CFR for SD at the end of the period was around 8%.

In tropical and subtropical regions, dengue is the most common arboviral disease. In this context, Colombia is an endemic region; however, incidence rates are variable within the country owing to the territories’ altitude. In 2019, the highest incidence rates, up to 5,809 cases per 100,000 population,^10^ occurred mainly in the center and south of the country in relation to the vector’s habitat, since *Ae. aegypti* does not circulate at altitudes higher than 2,200 m.a.s.l. In addition, dengue cases are reported throughout the year, and in the historical analysis, the high transmission season varies yearly. This dynamic is affected by various factors, including extreme climatic phenomena such as El Niño and La Niña and alterations in the normal circulation patterns of the ocean and the atmosphere, which change environmental conditions on a global scale. This climatic variability influences the population dynamics of the vectors and the extrinsic incubation period of the virus, affecting the incidence of dengue.^30^

Based on the PAHO database, DENV-1, 2, and 3 circulated in Colombia from 2017 to 2019, while from 2012 to 2016 and 2020, all four serotypes co-circulated. More precise information about the distribution of dengue serotypes in the country could contribute to the knowledge of the behavior of the infection. Although none of the studies identified reported national dengue seroprevalence, the regional data further highlight the hyperendemicity of dengue in Colombia. High seroprevalence rates in both children and adults and a correlation between increasing age and seroprevalence showed the extent of infection and latent risk in all population groups. Simultaneous IgM and IgG seropositivity could indicate secondary infections by any dengue serotype, which constitutes an increased risk of complications in the next event, particularly SD.

Currently, serological testing for dengue is challenging owing to the likelihood of cross-reactivity with other arboviruses. In addition, coinfections (chikungunya, and Zika) should be considered because of the increased risk of mortality, with serological diagnostic options that are not available in most of the country’s health institutions.^31^ In this scenario, that limits the correct epidemiological approach to arboviruses; a detailed clinical examination is a valuable tool with an adequate interpretation of the blood count, supporting early identification of patients at high risk of hematologic complications. However, the differential diagnosis of tropical fever, including leptospirosis, hantavirus, rickettsiosis, and malaria, is also challenging. Furthermore, according to epidemiological timing, dengue and COVID-19 infection are a syndemic that may increase the likelihood of adverse outcomes and the number of patients requiring intensive care and ventilatory support.^15^

Regarding the distribution of dengue cases by age in Colombia, the population under 20 years was the most affected. However, the overall number of people at risk is high, and other groups have been of great interest in recent years owing to the increase in frequency of cases. Although the disease has been considered of low frequency in older adults, ageing could increase the need to use hospital resources. The impact of dengue in particular subpopulations, such as immunocompromised and pregnant women, is a clinical challenge and requires special considerations.

More than 76% of patients with DWS were hospitalized in 2020.^15^ That same year, mortality due to confirmed dengue showed significant regional variability, ranging from 1.8% to 21.8% in the most affected areas of Colombia. These data show a high burden of disease associated with the number of people affected, the use of resources for hospital care of patients, and lethality. Just as the incidence of the disease varies across the country’s territories, clinical actions (diagnosis, treatment, and referral), access, resource availability, and quality of health services—the resources available for patient care—also differ by territory, with a probable impact on outcomes.

This review has several strengths and weaknesses. This was a comprehensive search of multiple sources that included grey literature and journal articles published in English and Spanish to minimize publication bias. However, the primary limitations and data gaps of this review are that 1) expansion factors for the dengue case definitions and from a national perspective were not reported and 2) the NIH did not report on the regional incidence of dengue by age and dengue seroprevalence. In addition, the data have inherent biases, which should be considered when interpreting the information. The WHO reports clinically suspected and laboratory-confirmed cases, whereas PAHO publishes the total number of suspected cases, which imposes differences with the INS reports, the main source of data. Also, sometimes there are data adjustments after being reported and even sent to PAHO and WHO, which increases or decreases the final number of cases. However, it is essential to note that the Ministry of Health is the primary reporting agency. Underreporting of dengue cases in the regions has been documented, related to several factors such as home management of mild cases and nonreporting of suspected cases by health professionals.^8^^,^^28^

## CONCLUSION

In conclusion, dengue in Colombia is a hyperendemic disease, with control measures that have been insufficient to prevent infection and the impact of the disease on the population. This scenario exposes the urgent need for cost-effective measures to control dengue, such as vector control and vaccination according to the epidemiological burden.^15^

Other approaches to dengue prevention, different from traditional strategies of limiting contact with the vector, will offer potential benefits to the population at risk, particularly to vulnerable groups, which are disproportionately affected by severe disease.

Entomological surveillance and predictive modeling are also crucial in high-risk national territories with the highest burden of dengue. In these areas, prioritization of education and prevention activities is critical. The support of local authorities, risk management entities, and healthcare institutions will be essential to align preventive actions with improvements in the availability, affordability, and timely use of laboratory tests.

Finally, dengue is a neglected tropical disease defined by the WHO, with devastating social, economic, and health consequences. Although progress has been made in dengue control in endemic settings, the clinical, financial, and use of health service burdens are considerable. Accordingly, dengue research and strengthening of prevention strategies must continue to reduce the risk and impact of dengue in Colombia.

## Supplemental Materials

10.4269/ajtmh.23-0907 Supplemental Materials
