# Supplementary material for: The Epidemiological Impact of Dengue in Colombia: A Systematic Review
Source: Am J Trop Med Hyg. 2024 Oct 29;112(1):182–8. doi: 10.4269/ajtmh.23-0907 (PMC11720756; doi:10.4269/ajtmh.23-0907)
Supplement: Supplemental Materials [file tpmd230907.SD1.pdf]

Supplementary appendix

Figure 1S. PRISMA 2020 Flow Diagram

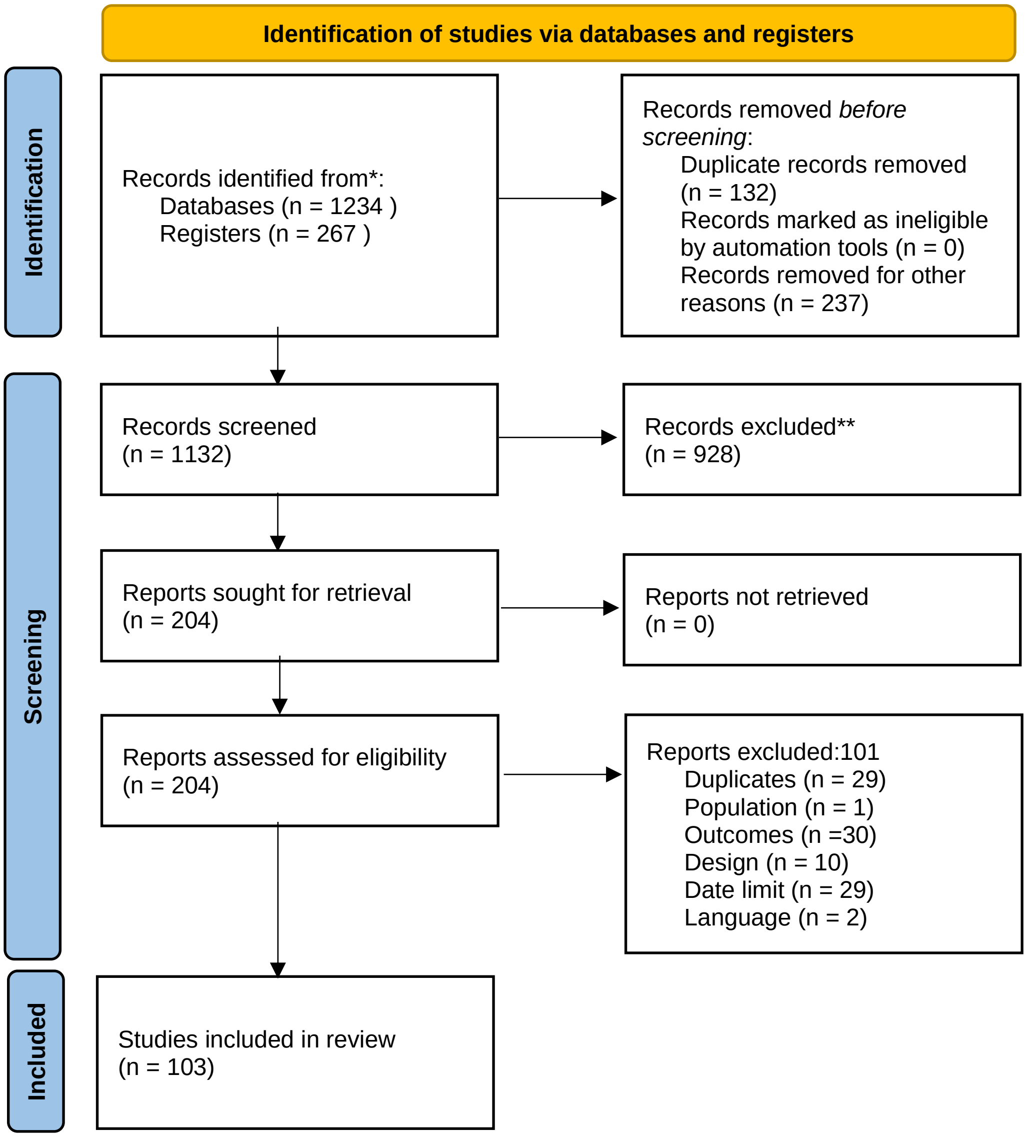

1. Epidemiology search strategies

EMBASE search strategy

Search line	Search terms
**Disease**	
1	Dengue/ or severe dengue/ or dengue virus/ or dengue haemorrhagic fever/ or dengue shock syndrome/ or dengue virus 1/ or dengue virus 2/ or dengue virus 3/ or dengue virus 4/
2	(Dengue or severe dengue or dengue virus or dengue hemorrhagic fever or alarm signs or DENV or DENV-1 or DENV-2 or DENV-3 or DENV-4).mp
**Epidemiology/clinical burden**	
3	Epidemiological studies/ or epidemiology/ or incidence/ or (epidemiology or incidence or frequency or risk or rate or proportion or cases or number or report).mp
4	Morbidity/ or mortality/ or mortality risk/ or mortality rate/ or fatality/ or comorbidity/ or disability-adjusted life year/ or (death or mortality or morbidity or case fatality rate* or case fatality ratio* or fatality rate* or case fatality risk* or severity or severe or lethality or hospitalization rate*).mp
5	(serotype/ or seroprevalence/ or seroprevalence or serotype or serotypes).mp or ((serotype* or serostatus) adj5 distribution*).mp
6	(expansion factor or expansion factors or underreporting factor).mp
7	(Primary infection or secondary infection or post-secondary infection).mp
**Combined results**	
8	1 or 2
9	3 or 4 or 5 or 6 or 7
10	8 and 9
11	10 and (Colombia).mp
12	**Limits applied to total search result***

*Total result in #11 limited to references published in English or Spanish from 2000 to 2020, and in human participants.

PubMed search strategy

Search line	Search terms
**Disease**	
1	dengue OR dengue virus OR dengue haemorrhagic fever OR dengue fever OR dengue infection ORDENV OR DENV-1 OR DENV-2 OR DENV-3 OR DENV-4
**Epidemiology/clinical burden**	
2	Epidemiology OR incidence OR frequency OR risk OR rate OR proportion OR cases OR number ORreport OR seroprevalence OR death OR mortality OR mortality rate OR case fatality OR case fatality rate OR morbidity OR lethality OR severe OR severity OR hospitalization OR comorbidity OR cross protection OR primary infection OR secondary infection OR post-secondary infection OR serogroup OR serotype OR serovar OR expansion factor OR expansion factors OR under reporting
**Country**	
3	Colombia
**Combined results**	
4	1 AND 2 AND 3
5	**Limits applied to total search results***

*Total result in #4 limited to references published in English or Spanish from 2000 to 2020, and in human participants only.

Grey literature sources

Sources	Link
WHO Library database (WHOLIS)	Dengue and severe dengue (who.int)
Pan-American Health Organization (PAHO)	PAHO/WHO | Pan American Health Organization
Instituto Nacional de Salud:<br>– SIVIGILA IQEN (Informe Quincenal Epidemiológico Nacional)<br>– BES (Boletin Epidemiologico Semanal)	http://www.ins.gov.co/Paginas/Inicio.aspx http://www.ins.gov.co/
Sistema Integral de Información de la Protección Social (SISPRO)	https://www.sispro.gov.co/Pages/Home.aspx
Thesis catalogue of:<br>· Universidad Industrial de Santander<br>· Universidad de los Andes<br>· Pontificia Universidad Javeriana<br>· Universidad del Rosario<br>· Universidad de Antioquia<br>· Universidad del Valle<br>· Universidad Nacional de Colombia	

* Last 3 years (2017-2020) of conference abstracts will be searched

2. Included studies' characteristics

Author (Year of publication)	Study design	Objectives	State/Location	Data sources	Data collection period	Population (number of patients/ sample size)	Male:Female Age	Limitations	Author conclusions
Lim (2012)	Cross sectional survey	to estimate the burden of dengue infection in both adults and children	Antioquia, Medellin	Residence from Medellin	nov-11	77	1- 55 years	NR	will be used to build comprehensive national investment cases of dengue vaccine in Thailand and Colombia.
Camacho (2014)	Cross sectional	To describe the frequency of circulation and phylogenetic characteristics of Dengue virus type 3, present in the department of Sucre	Sucre	NR	2013-2014	patients with febrile syndrome NR	All ages	NR	The results suggest that most of the dengue reported cases during this epidemic period were caused mainly by DEN3, but other two serotypes were present
Romero (2014)	Concordande study (Diagnostic test assessment)	To assess the validity of dengue fever reports and how they relate to the definition of case and severity.	Nationwide	Health institutions reports and SIVIGILA	2013	4359	F 40.4%, M: 59.6% All ages		Low concordance was observed between reporting and the review of clinical histories, which was associated with the low reporting of dengue fever compatible cases, especially milder cases.
Burgos (2015)	Space-time analysis	NR	Sucre: Sincelejo	seven districts of Sincelejo urban area	May to August 2014	NR	All ages		
Cardenas et al (2015)	Cross sectional	to estimate the age-specific dengue seroprevalence in the population of Piedecuesta, Santander, Colombia	Santander, Piedecuesta	Location in urban zone in Piedecuesta	July and October 2014	People living in 40 randomly selected locations in urban Piedecuesta. 1037	2 to 40 years	NR	These results show that Piedecuesta is an endemic area of DENV transmission, with large heterogeneities between urban and rural settings
Caribali (2015)	Cross sectional		Santander, Piedecuesta	SIVIGILA	2013	407	53% Female, Male: 47% 1-55 YEARS	NR	The data generated on dengue disease burden in Colombia would aid the policy makers for their decision making for upcoming dengue vaccine introduction in the country.
Castrillon (2015)		To analyze the incidence of dengue during the last ten years in Colombia (2004-2013), highlighting the periods and regions in which the largest number of cases was reported	Nationwide	SIVIGILA	2004 TO 2013	53141	All ages	NR	The results indicate that DENV infection presents a cyclic behavior that most likely will be repeated every three or four years and the occurrence of cases can be attributed to social as well as climate changes.

Pinzon (2015)	Retrospective cohort study	The objective was to assess 1997 and 2009 WHO classifications of severe dengue.	Bolivar: Cartagena	Hospitals	January 2013 to August 2014	Patients admitted with confirmed dengue test 761	m:50,6% f: 49,4% Median age was 7.9 years	NR	The WHO 2009 showed significant overperfomance compared WHO 1997
Castro (2016)	Burden of diseases	To estimate from 2010 to 2012 for the burden of the disease and the overall cost, calculated as the sum of medical costs, income lost owing to premature death, loss of productivity, and expenditure on direct, indirect, and prevention and monitoring activities for dengue infection in Colombia.	Nationwide	SIVIGILA And Colombian National Statistics Administrative Department	2010-2012	243042	All ages	nderestimation of both the burden of disease and costs.	the high burden of disease for dengue, as measured by DALYs, is confirmed in Colombia. Similarly, the economic costs of the disease are high and are largely borne by household
Chaparro (2016)	Observational descriptive	Describir el comportamiento de la mortalidad por dengue en Colombia entre 1985 y 2012.	Nationwide	National department of statistisc DANE	1985-2012	1990	M:55,6 , F: 44,4 All ages	Unprecise mortality rate due the numerator may be influenced by underreporting of deaths.	Dengue is an avoidable disease that should disappear from mortality statistics as a cause of death. The event is avoidable if the proposed activities from the Estrategia de Gestión Integrada (EGI)-Dengue are implemented and evaluated. We recommend encouraging the development of an informational culture to contribute to decision making and prioritizing resource allocation.
Coudeville (2016)	Mathematical model	to assess the population impact of dengue vaccination in the 10 countries participating in the phase III efficacy studies	Nationwide	surveillance of the two CYD-TDV phase III efficacy studies	2014	NR	All ages	As this data were collected from a clinical trial the participants must be ideal for the recruitment limitating the aplicability of the results	The analysis suggests that dengue vaccination can significantly reduce the public health impact of dengue in countries where the disease is endemic.
Fernandez (2016)	Observational, ecological study	To explore the annual seasonal dengue patterns in Colombia and in its five most endemic municipalities for the period 2007 to 2012, and for roughly annual cycles between 1978 and 2013 at the national level.	Nationwide	SIVIGILA		NR	All ages	Despite these findings, there was noa clear and continuous pattern that would suggest clear and consistent year-over-year seasonal pattern in all the years. No reports of all dengue	There were not conclusive patterns, patterns need to be explored at smaller aggregate levels, and their relationships with different predictive variables need to be investigated.

								cases, underestimating the real situation, Limitation on quality control data as a retrospective study	
Garcia (2016)	Retrospectiv e study	To describe mortality by dengue, socio-demographic characteristics and possible determinants associated to mortality in department of Meta.	Meta	SIVIGILA	January 2010 to May 2014	34	M: 18 , F: 17 All ages	NR	People younger than 10 years old, with scarce resources and from the subsidized system have a greater risk of mortality. Abdominal pain and vomit are important predictors of shock, it was also evidenced that the use of Aines (Spanish acronym for non steroidal anti-inflammatory agents) augments the risk, tests of liver function presented the greatest alterations, and hemoconcentration is not used to evaluate the risk of severe dengue.
Hernandez (2016)	Observationa l descriptive, retrospective	To characterize cases of dengue reported from 2009 to 2013 in Valle del Cauca department, Colombia, and to establish a methodology to develop endemic channels that can be applied to this event.	Valle del cauca	Regional database of health deparment	2009-2013	63655	F: 29771, M: 33884 All ages	NR	there were some years in which the number of cases was very low and others in which the epidemic reached very high levels.
Krystosik (2016)	Cross sectional	to better understand the epidemiological relationship between these diseases spread by a common vector.	Valle del cauca, Cali	SIVIGILA	October, 2014 and April, 2016	21210	All ages	Limitations of this study include the use of secondary passive surveillance data to estimate disease burden. We did not have access to laboratory confirmation for disease state. The sample was biased due to non-random underreporting, perhaps related to poverty and education.	Dengue incidence was higher than expected in weeks 3-10 of 2016. This increase may be due to increased reporting due to the Zika outbreak
Lazou (2016)		To provide a sample for epidemiologic analyses of symptomatic dengue in	Nationwide	Multiples center in Latin america	June 2011-	Children from the placebo	Male: female ratio: 0,97 9 - 18 years	This study included high incidence countries so	Burdens varied widely according to country, but the rates were generally higher and the disease

		children across 10 countries in Southeast Asia and Latin America in which dengue is endemic.			April 2014	group of Sanofi Pasteur developed a recombinant live, attenuated, tetravalent dengue vaccine (chimeric yellow fever–dengue–tetravalent dengue vaccine [CYD-TDV]). 6939 (3245 In Colombia)		generalization of these findings to other countries in the region may be limited, just healthy children were included whose parents were willing to have their children participate in a vaccine trial	more frequently severe in Asian countries than in Latin American countries.
Matta (2016)	Retrospective study	To describe the clinical profile of patients with dengue virus infection hospitalized in a tertiary hospital in the city of Cali, Colombia. We also describe the trend analysis of the number of cases by epidemiological weeks in 2013	Valle del cauca, Cali	Rafael uribe uribe clinic at Cali	2013	Febrile patiens with virologically confirmed dengue with IgM elisa 287	Male:female ratio 1:12 All ages		
Moreno (2016)	Cross sectional	Quantifies the compliance of notification concerning to probable cases of dengue in the West Metropolitan Area in 2014.	Risaralda	SIVIGILA and Individual reports (RIPS)	2014	549	All ages	It was limitated to a few institutions	Evidence of deficit in notification
Rodriguez (2016)	Cross sectional	To characterise the clinical and laboratory findings on a series of febrile cases with a presumptive diagnosis of dengue virus infection in Quindío, Colombia.	Quindio	Local health centers	Jan to Jul 2013	Febrile people suspected of dengue cases 149	All ages	NR	Dengue was confirmed as an important aetiology of acute febrile icterohaemorrhagic syndrome in Quindío
Rosso (2016)	Observational descriptive	To describe the prevalence and clinical course of dengue infection in elderly patients who were admitted to a referral care center for infectious diseases in an endemic region.	Valle del cauca, Cali	Clinic "valle de lili"	Jan 2011 to Dec 2014	Febrile ederly patients 37	M:19, F:18 >65 years	Quality of the information as a retrospective study	We found dengue infection to be more frequent than expected in this sample of elderly patients, due to acute febrile syndrome. Elderly patients also required higher rate of hospitalization and had more complications, however there were

									no deaths due to good management.
Sarti (2016)		to examine the background incidence of dengue.	Nationwide	CYD15 Study, National Epidemiological Surveillance System	June 2011 to April 2014	Healthy children 3245	9-16 years	Data obtained from a cohort study undertaken at selected geographical settings in a selected healthy paediatric population were compared with national data, with differents features	There is a substantial under-reporting of dengue in the NESSs. Understanding the level of under-reporting would allow more accurate estimates of the dengue burden in Latin America.
Sheppard (2016)	economic burden of disease	To estimate the global economic burden of dengue by country and super-region with associated uncertainty intervals (UI).	Nationwide	Global nurden analysis	2013	NR	All ages	Data not precise to countries due they are extrapolation data	Our estimates indicate that dengue illness imposes costs greater than other major infectious diseases with comparable data such as cholera, rotavirus, gastroenteritis, chagas.
Stanaway et al (2016)	Observational study	To estimated dengue mortality, incidence, and burden for the Global Burden of Disease Study 2013.	Nationwide	The Global Burden of Disease Cause of Death database	2013	510100	All ages	Limited information about age specific data on the incidence of dengue	The study results offer more evidence that the true symptomatic incidence of dengue probably falls within the commonly cited range of 50 million to 100 million cases per year. Mortality rates might be higher
Bello (2017)	Mathematical model	to estimate the Cohen's Kappa measure of agreement for the interpretation of the change in relative risk of dengue between the global and annual time scales.	Santander, Bucaramanga	SIVIGILA	January 2008 to December 2015	27301	All ages	to find quality data for generating expected values as input for the models.	The study provides an example of implementation of relative risk estimation using Bayesian models for disease mapping at small spatial scale with covariates
Bracho (2017)	Cross sectional study and cohort study	to learn the felt demand for dengue vaccines by estimating the willingness to pay and its associated factors in endemic communities of the North Caribbean region of Colombia.	La Guajira (Riohacha, Albania, Fonseca, San Juan del Cesar, Distracción, Maicao, Villanueva, Uribia and Manaure) one in Cesar (Valledupar) and one in Magdalena (El Retén)	11 municipalities in the Colombian caribbean	October to December 2015	Residents from the municipalities 1037	F:85% , M :15% All ages	does not refer to any particular available vaccine.	Factors such as price, number of doses and effectiveness can independently influence the decision to purchase a vaccine against an endemic disease, such as dengue
Carabali et al (2017)	Prevalence survey	To describe the dengue serological prevalence and the seroconversion rate of individuals between 1 and	Antioquia, Medellin	12 neighborhoods of Santa Cruz	November 2011 to February 2014	Healthy residents 3684	F:2450 M:1234 1-65 years	The authors did not considered the introduction of chikungunya and	Te study evidenced permanent and ongoing dengue

		65 years of age in Medellin using a tiered dengue serological survey approach.		Comuna, medellin city				Zika , it conducted prior of it; the presence of heterotypic responses might limit the interpretations about the serotype-specific incident cases.	
Fuentes (2017)	Space-time analysis	To characterized the spatial and temporal behaviours of dengue in Girardot and discusses the potential territorial dynamics related to the distribution of this diseas	Cundinamarca, Girardot	SIVIGILA and census data		2012 to 2015 2027	M: 52.1%, F: 47,9% All ages	Underreport of dengue cases, lack of information about dengue serotypes	Dengue did not show linear association with poverty or with vulnerable peripheral spaces in intra-urban settings
Jimenez (2017)	Cross sectional	To establish the seroprevalence of dengue infection in neighborhoods with high incidence in the municipalities of Armenia, Calarcá, La Tebaida and Montenegro, Quindío, in 2014.	Quindio: Armenia, Calarcá, La Tebaida y Montenegro	Neighborhood selected from the cities included: Armenia, Calarcá, La Tebaida y Montenegro	2014	Healthy volunteers 658	f:447, m: 211 All ages	NR	The study found a high prevalence of both IgG and IgM in the four municipalities. We had positive results for IgM in all age groups, which suggests recent infection. We also found simultaneous seropositivity for IgG and IgM (12.9%), which may indicate infection by another serotype or presence of infection in the past three months. A multisectoral approach is necessary for dengue control in Quindío.
Montoya (2017)	Cross sectional	to study the relationship between dengue cases and the variables given by the social, geographic and economic data of the 23 municipalities of the department of Atlantico	Atlantico	SIVIGILA	2010-2013	7390	All ages	The study is based on the reported cases in just one deparment from Colombia	it was found that the variables related to the social characteristics present in the dwellings and the disordered growth of the urban center were those that had the greatest influence In the increase of the number of dengue cases. The Bayesian model allowed the identification of the relationship of dengue with factors outside the health sector, establishing areas of higher risk of disease.
Padilla (2017)	Observational descriptive	To determine the behavior and transmission scenarios of vector-borne diseases in Colombia between 1990 and 2016.	Nationwide	SIVIGILA, morbitisy database SISPRO, national department of statistic DANE	1990-2016	DENGUE CASES 1401240	All ages	Information quality as a retrospective study and not all dengue cases were laboratory confirmed.	Dengue is an endemic disease in Colombia, with different transmission patterns

Rabba (2017)	Posthoc genomic Study	to document the genetic distance between the components of the CYD-TDV formulation and the DENV strains detected amongst cases in the CYD14 and CYD15 trials.	Nationwide		June 2011 and April 2014	664	All ages	The study was not aimed to detect dengue serotype in Colombia, small sample size as a poshoc study	
Sanchez (2017)	Mathematical model	To assess the effect of climatic variability on the dynamics of dengue fever transmission in the Department of Cauca	Cauca	SIVIGILA of Colombia and National Institute of Hydrology, Meteorology, and Environmental Studies of Colombia (IDEAM).	January 2006 to December 2015,	4464	All ages	NR	Dengue cases increased during the period of study with higher presence during the months with El Niño phase. The mean temperature had a higher influence than precipitation on dengue cases
Sanchez (2017)	Cross sectional	to determine the frequency of Dengue infection in patients with acute febrile syndrome in Meta's Administrative District, Colombia.	Meta, Villavicencio	SIVIGILA and Hospital	May 2013 , Jun 2014	Febrile dengue case 100	F:51, M:49 5 and 65	No sample was taken, extrapolation problems due small sample	Dengue virus is prevalent in this area,
Carabali (2018)	Prospective surveillance study	To estimate the age-specific incidence of symptomatic dengue and chikungunya in Colombia.	Santander, Bucaramanga	Hospitals	August 2014 to August 2015	839	M:422, F: 417 1- 55 years	Losses in the follow up, inferences about asymptomatic or mild cases should be limited, no conclusions for people over 56 years	These findings highlight the ongoing coexistence of both arboviruses, a distinct clinical profile of each condition in the study area that could be used by clinicians to generate a differential diagnosis, and the presence of underreporting, mostly among hospitalised cases
Carrillo (2018)	Cross sectional	to use molecular methods to identify their co-circulation as well as the prevalence of co-infections, in a cohort of patients at the Colombian-Venezuelan border	Norte de santander: Villa del rosario	Hospitals	August 2015 to April 2016	157	M: 54, F:103 All ages	NR	This study demonstrate the simultaneous co-circulation of DENV, CHIKV, ZIKV and their co-infections at the Colombian-Venezuelan border. Moreover, it is necessary to improve the differential diagnosis in patients with acute febrile syndrome and to study the possible consequences of this epidemiological overview of the clinical outcomes of these diseases in endemic regions.
Castro bonilla (2018)	Cross sectional	to identify the factors associated with DENV infection and reinfection in a group of school children	Cundinamarca, Anapoima and Apula	Anapoima and Apulo area	2014	Asyptomatic children 347	F:176 , m : 171 4-14 years	It was not possible to establish causal associations;	Risk factors evaluation showed that children aged 8 years and older living in the municipalities for more than 7 years were more

		from 4 to 14 years old in these two Colombian municipalities.						courtesy bias, information bias	likely to be infected or reinfected by DENV. In the same way, poor nutrition, lack of water supply, sewer service, or waste disposal services could raise the likelihood of dengue infections
Desjardins (2018)	Mathematica l model	to identify and compare statistically significant space-time clusters of CHIK and DENF in Colombia during the outbreaks of 2015 and 2016	Nationwide	SIVIGILA	2015 to 2016	77773	All ages	First, a cluster's relative risk reported by the scan statistic does not consider sociodemographic variations within the radius.	The paper successfully identified significant univariate and multivariate space-time clusters of DENF and CHIK in Colombia during the outbreaks in 2015 and 2016, while discussing some of the processes which were responsible for the epidemics
Hernandez (2018)	Retrospectie study	Determine the epidemiological behavior and the geographic distribution of dengue fever casestreated in the San Rafael Hospital in the municipality of the El Espinal, from 2010 to 2014.	Tolima: Espinal	SIVIGILA reports from Hospital San Rafael	2010-2014	3264	M:1758, F: 1506 All ages	not possibility of control the initial collection of data or the correct processing of them forms.	The results found are above local, national and international parameters on incidence, mor-tality, and case-fatality. Through the analysis of endemic behavior and geographic distribution, times andspecific areas are provided to optimize public health measures
Jiménez-Silva et al (2018)	Retrospectiv e, genomic study	To provide insights into the evolution and population dynamics of all four dengue serotypes in the region of Santander	Santander, Bucaramanga	Laboratory of Arbovirus, Tropical Diseases Research Center (CINTROP)	1990-2015	193	All ages	Most of the samples were collected from a single region and this limits our knowledge on the country-wide viral genetic diversity.	The study documented the co-circulation of a single genotype of each serotype, and the unapparent circulation of every serotype prior its first detection in the country. This study also showed that genetic diversity of serotypes circulating in the country continues to grow due to in-situ evolution and recurring introductions of viral strains from different countries in the region.
Kristosik (2018)	Retrospectiv e study		Valle del cauca, Cali	SIVIGILA	October 2014 to April 2016	21210	All ages	NR	Patterns in incident cases emerged over time with clear outbreak seasons and concurrent outbreaks and over space in high risk neighborhoods.
Krystosik (2018)	Retrospectiv e study	To analyze patterns of arboviral risk and access to laboratory diagnostics	Valle del cauca, Cali	SIVIGILA	2014 to 2016	21210	All ages	NR	Crucially, an overall higher risk of homicide is associated with increased risk of reported DENV, lower rates of acute testing, and higher rates of lab versus clinical discordance.
Martinez Bello (2018)	Mathematica l model	to estimate the parallel relative risk of Zika virus disease (ZVD) and dengue	Santander, Bucaramanga	SIVIGILA	October 2015 to	7891	All ages	For dengue data, it is well known that underreporting	This study thus presents a helpful statisticaL method as a tool for decision making in preventing and

		using spatio-temporal interaction effects models for one department and one city of Colombia during the 2015–2016 ZVD outbreak.			Decembe r 2016			could reach almost 70%, because many affected people do not approach health services and some of the patients attending the health services are not correctly diagnosed	controlling vector-borne emergent diseases
Nouvelle 2018 (2018)	Cross sectional		Norte de santander, Sucre, Antioquia (sector of the city of Medellín), Cúcuta, Neiva, Sincelejo		October to Decembe r 2016	2400	All ages	NR	DENV seropositivity with age confirmed the endemic nature of its transmission, whereas exposure to both ZIKV and CHIK appeared unrelated to age confirming their epidemic nature.
Pasin (2018)	Prospective study	To evaluate the association of rainy season with overall dengue disease incidence and with the efficacy of the Sanofi Pasteur recombinant, live, attenuated, tetravalent vaccine (CYDTDV) in two randomized, controlled multicenter phase III clinical trials in Asia and Latin America.	Nationwide	Hospital	From June 2011 to Dec 2013	Children 25824	M:12683, F: 13141 9-16 years	NR	Although dengue transmission and exposure are expected to increase during the rainy season, our results indicate that CYD-TDV vaccine efficacy remains constant throughout the year in endemic regions.
Piedrahita (2018)	Longitudinal study	to estimate DENV transmission in schoolchildren population (aged 5–19 years) in Medellin, Colombia.	Antioquia, Medellin	Schools	May 2010 to May 2012,	schoolchildre n enrolled at any educational stage 799	F: 52,9%, M: 47,1 % 5 - 19 years	NR	This study revealed high DENV transmission in schoolchildren determined as "sentinel population." High DENV risk was found in districts with combined poorly socioeconomic conditions and densest human and mosquito populations
Tarantine (2018)	Cross sectional	To evaluate the case report forms and times elapsed between the surveillance steps for dengue virus (DENV) infection in a large Colombian city before the emergence of other arbovirus epidemics	Atlantico, Barranquilla	Hospital	2009 - 2013	Physicians who attends dengue cases 8115	M:4411, F: 3704 All ages	NR	The system was accurate, simple, flexible, stable and acceptable, but a number of ways are suggested to improve this case detection and reporting system.
Tuesca (2018)	Cross sectional	estimate the sero-prevalence of IgG and IgM antibodies for dengue and associated factors.	Atlantico, Barranquilla	Area of Barranquilla	March to May 2014	Residents from Barranquilla 478	All ages	NR	The high sero-prevalence for IgG is explained by circulation of the four serotypes in the country, epidemic outbreaks every two to three years, and unfavorable behavior

									and practices related to water management.
Zeng (2018)	Cost effectiveness study	To evaluate the cost-effectiveness of dengue vaccination in populations similar to those at the trial sites in those same Latin American and Asian countries.	Nationwide	results of the two large-scale phase-3 efficacy trials.	30 years	NR	All ages	Clinical trial does not provide direct data on the magnitude of the herd effect against dengue transmission	Vaccine could have a favorable economic value in sites similar to those in the trials.
Cardenas et al (2019)	Cross sectional and cohort study	Present preliminary findings from our population based studies conducted in Piedecuesta, Colombia between 2014 and 2017 around the time of chikungunya (CHIKV) and Zika (ZIKV) emergence.	Santander, Piedecuesta	Population	2014 to 2017	2400	All ages	NR	
Carreño (2019)	cross-correlation analyses	To investigate the link between fluctuations in the prevalence of dengue virus (DENV) serotypes and the number of dengue cases in the metropolitan area of Bucaramanga, Santander State, Colombia, in the 2007–2010 and 2014–2017 periods.	Santander, Bucaramanga	SIVIGILA and local laboratory	2007–2010 and 2014–2017 periods	2009-2010: 204 and 2014–2017:2293	All ages	The few DENV isolates did not represent the viral population circulating in the metropolitan area of Bucaramanga.	This is the first study that links serotype dynamics and dengue incidence in Colombia. In the last 10 years, DENV-1 and DENV-2 have been the dominant serotypes across the country followed by DENV-3, whereas DENV-4 has remained low.
Lopez montenegro et al (2019)	Time series study	To forecast the number of dengue cases expected in Colombia from 2018 through 2022	Nationwide	National Institute of Health routine surveillance system database - SIVIGILA	February 2017 to March 2018	NR	All ages	NR	The study predicted the highest incidence of dengue cases in Colombia would occur in 2019.
Martínez-Bello et al (2019)	Observationa l retrospective study	To estimate the disease- and area-specific RRs for dengue and Zika	Santander, Bucaramanga	SIVIGILA	2015-2016	7891	F: 48,6%, M: 51,4% All ages	NR	Information on the association between diseases is of value, especially in areas where multiple arboviruses cocirculate, and can be used to improve inferences and interpretations and thus contribute to informed public health decision making
Mercado (2019)	Cross sectional	to determine the frequency of coinfection in samples processed by the Arbovirus Laboratory of the Colombian National Institute of Health and to	Nationwide	SIVIGILA	October 2015–Decembe r 2016	23871		Retrospective design of the study, 2. Also, it is possible that other cases were not reported to the SIVIGILA. 3.	the frequency of arbovirus (DENV, CHIKV and/or ZIKV) coinfection during the ZIKV epidemic in common.

		describe the mortality cases associated with coinfection that occurred during epidemiologic surveillance of the zika epidemic in Colombia.						immunohistochemical and serological studies were not performed	
Rico Mendoza (2019)	Cross sectional	This study aimed to identify the co-circulation patterns of three viruses (dengue, Zika, and chikungunya) in Colombia from 2008 to 2018 by using notification reports provided to the national surveillance system.	Nationwide	SIVIGILA	2016	101016	Male 50%, female 50% All ages	Underreporting dengue cases	The decrease in the number of dengue cases after co-circulation of the three viruses could indicate possible cross-protection. This finding should be further analyzed
Rojas (2019)	Case/control study	To provide evidence that features of severe dengue, including clinical and laboratory parameters that can be easily assessed early during the course of the disease, are actually associated with higher mortality in dengue patients.	Valle de cauca and Santander	Hospitals	2009 to 2015	110 cases and 217 controls	F:63%, M: 57% All ages	NR	older age, past medical history with emphasis on previous hospitalization, along with basic physical examination measurements such as heart rate and blood pressure determined early on the course of dengue infection might be used in the stratification of hospitalized patients regarding their risk of death.
Rosso (2019)	Retrospective study	to describe trends in dengue cases and mortality in the ICU for 15 years in Cali, Colombia.	Valle del cauca, Cali	Clinic Fundacion valle de Lili	2012-2015	Dengue cases treated in ICU 70	M:54%, F:46% All ages	Limited information	Dengue mortality cases in the ICU have decreased in the last 15 years, which is related to early admission to the ICU and continuous clinical monitoring.
Salvo (2019)	Cross sectional	to provide a precise characterization of viral diversity throughout seasons of overall serotype dominance and waning prevalence.	Antioquia, Bello	dengue-suspected human serum	June of 2014 to July of 2016,	Dengue suspected human 990	All ages	Small sample considering just a specific region of colombia	these data suggest that the NS1 gene, rather than the E gene, may be a target of positive selection, although not mutually exclusive, and potentially useful sentinel of adaptive changes at the population level.
Triana (2019)	Observational descriptive	to describe the spatial and temporal distribution of dengue, zika and Chikungunya viruses in Colombia and to identify clusters at spatial, temporal and space-temporal levels.	Nationwide	SIVIGILA	2006-2017	782353	All ages	NR	Two conglomerates were confirmed for the Dengue, Zika and Chikungunya events, that could be established as areas of higher risk of co-infection
Aviles vergara et al (2020)	Cross sectional	To determine simultaneous circulation of DENV serotypes and ZIKV	Cordoba: 5 municipalities (monteria, cerete,	Hospitals	June 2015 to	Febrile patients with clinical	f:150, m: 144 All ages	Possible bias in the sample selection	We reported the co-circulation of all four DENV serotypes, with a higher frequency of DENV-2

		in Córdoba, Colombia, during 2015 and 2016	planeta rica, montelibano y sahagun)		Decembe r 2016	diagnosis of suspeted dengue 294			
Caceres (2020)	Cross sectional	to estimate the prevalence of DENV, ZIKV, and CHIKV infections in blood donors from endemic and non-endemic areas attending Colombian Red Cross Blood Banks.	Nationwide	Hospital	EW 47 2019 to 4 2020	All ages 311	All ages	NR	we detected a high prevalence of blood bank donors infected with DENV, ZIKV, or CHIKV
Cardenas et al (2020)	Cross sectional	to estimate the age-specific seroprevalence and transmission intensity of DENV and to characterize risk factors for infection.	Santander: Bucaramanga, Piedecuesta	Area of Piedecuesta	July to October 2014	1283	F: 758, M:525 2-40 years	Sample was probabilistic, rural samples were obtain from a single "vereda" that was chosen based on its accesibility	Long term endemic circulation of DENV in Piedecuesta, with large heterogeneities between urban and rural areas located just a few kilometers apart.
Desjardins (2020)	Mathematica l model	to examine the influence of socioeconomic, environmental, weather, and climatic variables on DENF outbreaks in Cali, Colombia neighborhood and weekly levels between 2015 and 2016	Valle del cauca, Cali	Colombia's National Institute of Health and the Global Historical Climate Network archive	2015-2016	NR	All ages	underreporting of cases and unmatched addresses during the geocoding process likely undermines the true burden of DENF	The temporally lagged weather covariates can significantly estimate when risk of transmission is highest, and the spatial covariates can help explain the differences in disease risk at the neighborhood level. Adding weather and climate data to a space–time model can improve disease surveillance, especially for VBDs that require specific conditions for transmission to occur.
Hernandez (2020)	Longitudinal study	to assess the epidemiological trends and exploratory disease burden indicators of four infectious diseases: dengue, tuberculosis, zika and chikungunya in Colombia.	Nationwide	Public health surveillance system (SIVIGILA)	2014 - 2018	NR	All ages	Lack of total number due the publication type	Real-World Data (RWD) can be a powerful tool for early outbreak detection and healthcare resource allocation for tropical diseases in Latin America.
Lim (2020)	Prevalence surveillance study	To understand epidemiology and genetic diversity of circulating DENVs, in a catchment area population of approximately 100,000 residents in Medellin, Colombia	Antioquia, Medellin	Hospitals	Novembe r 2011–February 2014	537	F:313 M:224 All ages	1. considerable dnegue burden with the majority mild illness , 2. the study findings support genetic diversity of DENV isolates and their relatedness to isolates from other nearby countries	Thi study is under-ascertainment of the community residents with relevant symptoms

Marban (2020)	Cross sectional	to assess the seroprevalence of ZIKV, DENV, and CHIKV infections among pregnant women, in an area of South America where these arboviral infections co-exist.	Cordoba: Cerete	Local health center	may-16	Pregnant women with symptons compatible with arboviral disease 90	All female All ages	Small sample size, selection bias of participants could occur, as only those who were aware of this call, by already attending antenatal care services or having a radio, could find out information of the study to attend the health center. 2. plaque reduction neutralization tests were not available to confirm serological results, and molecular diagnosis was not performed; thus, minimizing the chances of detecting active arboviral infection.	Pregnant women had a high seroprevalence of past arboviral infections to Zika, Dengue, and Chikungunya.
Molineros (2020)	Cross sectional	To determine the seroprevalence of dengue in five municipalities of Valle del Cauca with hyperendemic and mesoendemic transmission of the disease	Valle del cauca: Cali, Palmira, Buga, Tuluá y Cartago	From a projet ran by the Health ministery	2014	Residents from those municipalitie s 822	f:65,8%, M : 34,2% All ages		The high seroprevalence of dengue in Valle del Cauca shows the impact of this disease in the life history of the residents
Quintero (2020)	Ecological study	To present the impact of an Aedes-vector control intervention "Girardot Aedes-free" in reducing the number of reported dengue cases in Girardot, Colombia, between 2015 and 2017.	Cundinamarca, Girardot	Communicable Disease Surveillance System of Girardot, SIVIGILA	March 2015 to August 29 2017	702	M: 52,9% F: 47,1% All ages	The surveillance system only captures symptomatic patients who sought treatment at health care services, and are registered with a residential address that is not necessary the location of dengue transmission. No specific information for serotype	The results indicate a reduction in dengue incidence compared to matched controls sites, although this is probably an underestimated of the true potential of the intervention.
Reynales (2020)	Posthoc Case–Cohort Study	To describe the efficacy and safety of CYD-TDV in participants from	Armenia, La Tebaida, Montenegro,	Hospitals	June 2011 to	Children 959	9-16 years	Data on severe dengue and dengue hospitalization are	In Colombia, where seroprevalence has been demonstrated to be high in several regions of the country,

		Colombia, based on the data from CYD15.	Calarcá, Girardot, Yopal, Aguazul, Acacías and Bucaramanga		March 2018			limited due to the relatively low number of hospitalized and/or severe confirmed dengue cases	CYD-TDV is a useful tool to consider as part of an integrated control strategy against endemic dengue, a disease with a high economic impact on the health system.
Rojas (2020)	Case-control study nested in a cohort	To identify the clinical manifestations and demographic characteristics associated with severe dengue in a pediatric institution in Cali, Colombia, between January 2015 and December 2016	Valle del cauca, Cali	Fundacion Club Noel	Jan 215-Dec 2016	Hospitalized patients with dengue 200	F: 53%, M: 47% <18 years	NR	Dengue in the pediatric population has a nonspecific clinical behavior, however, identifying pleural effusion and cardiovascular alterations can explain an admission to PICU and warn of a possible fatal outcome
Vasquez (2020)	retrospective longitudinal study	to describe the economic burden of dengue epidemics between 2010 and 2016, in the State of Meta, Colombia	Meta	SIVIGILA	2010-2016	652221	All ages	the incorporation of national estimated values for the cost of outpatient and inpatient medical care. No uso of tecniques for the adjustment of underreported dengue cases	dengue is a disease with high economic burden in the state of Meta (USD 204,819,006 in 2011 – USD 1,615,456,216 in 2013)
Velandia (2020)	Prevalence study	to determine the prevalence of dengue IgM and IgG antibodies in healthy children and adults in urban and rural areas of seven different endemic regions in Colombia between 2013 and 2015.	Cundinamarca (anapoima, apulo), Nariño (tumaco), Valle del cauca(buenaventura), Choco(quibdo), Cordoba (tierralta), Sucre (Sincelejo)	Home location	April 2013 to October 2015	healthy children and adults in urban and rural areas across different endemic regions in Colombia 1318	F:783, M: 536 All ages	possible cross-reactivity of immunoglobulin tests between arboviruses yellow fever virus and ZIKV.	This study confirmed the broad and permanent circulation of DENV in Colombia and the high rates of infection and reinfection suffered by its inhabitants
Carabali (2021)	Cross sectional	To examine (scope, nature and degree of) underreporting of chikungunya, dengue, and Zika case data in the national surveillance system of Cali, Colombia from 2014-2017.	Cali, Medellin, Villavicencio	national disease surveillance program, SIVIGILA, health facilities	2014 to 2017	266549	All ages	NR	There are limitations to our study including the lack of information obtained on laboratory confirmation of cases, resulting in limited sensitivity and specificity information. Importantly, some of the healthcare facility data were incomplete and prevented us from a more comprehensive analysis with additional covariates. For instance, detailed information on symptoms, complete blood counts reports, and treatment received

									could have been used to identify severe arboviral cases and the characterization of reporting by age was not possible due to incomplete individual age data from one healthcare facility. Given the quality of the data, the results were restricted to clinically confirmed diagnosis of arbovi-ruses at healthcare facilities. For Medellin and Villavicencio, it was not possible to conduct stratified analysis by healthcare provider given the absence of information about the type of insurer or because the healthcare facility provided care for different insurance schemes. Finally, for the MC-SIMEX analysis we used three correlation matrixes for the simulation, however, there are several other combinations that could have been used for the correction, each one resulting in different estimates of misclassification
Cardona-Ospina et al (2021)	Observational, ecological study	To study the current epidemiological trends for both diseases (dengue and COVID-19) in Colombia at a district and departmental levels (primary administrative level), as well as nation-wide	Nationwide	Public epidemiological surveillance system online	2015 - 2020	452980	All ages	NR	As both COVID-19 and dengue may lead to fatal outcomes, especially in patients with chronic co-morbidities, overlapping infections and their co-occurrence may increase the number of patients requiring intensive care and mechanical ventilation. Intensified preparation for such scenarios should be considered
Castellanos et al (2021)	Prospective study	To identify the arbovirus involved in febrile cases identified in a pediatric clinic in Cali, Valle del Cauca province, Colombia, and study the clinical characteristics.	Valle del cauca, Cali	Clinic "Club Noel"	July 2018 to August 2019.	Children with symptons such as fever or two symptoms such as rash, edema, headache, myalgias, or arthralgias 345	M: 179, F: 166 <18 years	1. The population is not representative, 2. There were not an external validation for the PCR test	CHIKV circulation in this endemic area causing mild and severe febrile disease in children

NR not reported; SIVIGILA Public Health Surveillance System

3. Quality assessment

	Cardona-Ospina 2021	Stanaway 2016	Jiménez-Silva 2018	Carabali 2017	Martínez-Bello 2019	Cardenas 2020	López Montenegro 2019	Castellanos 2021	Avilés-Vergara 2020	Velandia 2020	Martinez Bello 2018	Lim 2020
Was the research question or objective in this paper clearly stated?	Yes	Yes	Yes	Yes	Yes	Yes	Yes	Yes	Yes	Yes	Yes	Yes
Was the study population clearly specified and defined?	Yes	Yes	Yes	Yes	Yes	Yes	Yes	Yes	Yes	Yes	Yes	Yes
Was the participation rate of eligible persons at least 50%?	Yes	Yes	Yes	Yes	Yes	Yes	Can't tell	Can't tell	Yes	Yes	Cant tell	Yes
Were all the subjects selected or recruited from the same or similar populations (including the same time period)? Were inclusion and exclusion criteria for being in the study prespecified and applied uniformly to all participants?	Yes	Yes	Yes	Yes	No	Yes	NA	No	Yes	Yes	Yes	Yes
Was a sample size justification, power description, or variance and effect estimates provided?	No	No	No	Yes	No	Yes	NA	No	No	Yes	No	Yes
For the analyses in this paper, were the exposure(s) of interest measured prior to the outcome(s) being measured?	No	No	No	Yes	No	No	NA	No	No	No	NA	No
Was the timeframe sufficient so that one could reasonably expect to see an association between exposure and outcome if it existed?	No	No	No	No	NA	No	NA	Yes	No	No	NA	No
For exposures that can vary in amount or level, did the study examine different levels of the exposure as related to the outcome (e.g., categories of exposure, or exposure measured as continuous variable)?	NA	Yes	NA	Yes	NA	Yes	NA	Yes	Can't tell	Yes	NA	Yes
Were the exposure measures (independent variables) clearly defined, valid, reliable, and implemented consistently across all study participants?	No	No	Yes	Yes	Yes	NA	NA	No	Yes	Yes	No	Yes
Was the exposure(s) assessed more than once over time?	NA	NA	NA	NA	NA	NA	NA	No	No	No	No	No
Were the outcome measures (dependent variables) clearly defined, valid, reliable, and implemented consistently across all study participants?	Yes	Yes	Yes	Yes	Yes	Yes	Yes	Yes	Yes	Yes	No	Yes
Were the outcome assessors blinded to the exposure status of participants?	NA	NA	No	No	No	No	NA	No	No	No	NA	No
Was loss to follow-up after baseline 20% or less?	NA	NA	NA	No	NA	No	NA	No	NA	NA	NA	No
Were key potential confounding variables measured and adjusted statistically for their impact on the relationship between exposure(s) and outcome(s)?	NA	Yes	Yes	Yes	Yes	Yes	Yes	Can't tell	Yes	NA	NA	No
Final score: over 70% (green), 50-70% (amber) and less than 50% (red)	55,56%	63,64%	63,64%	76,92%	60,00%	58,33%	36,36%	35,71%	53,85%	58,33%	37,50%	57,14%

	Rojas 2019	Chaparro 2016	Bello 2017	Carabali 2018	Castro bonilla 2018	Carreño 2019	Krystosik 2018	Carrillo 2018	Reynales 2020	Desjardins 2018	Desjardins 2020	Jimenez 2017
Was the research question or objective in this paper clearly stated?	Yes	Yes	Yes	Yes	Yes	Yes	Yes	Yes	Yes	Yes	Yes	Yes
Was the study population clearly specified and defined?	Yes	NA	Yes	Yes	Yes	Yes	Yes	Yes	Yes	Yes	Yes	Yes
Was the participation rate of eligible persons at least 50%?	Cant tell	NA	Cant tell	Cant tell	Yes	Cant tell	Cant tell	Yes	Yes	Yes	Yes	Yes
Were all the subjects selected or recruited from the same or similar populations (including the same time period)? Were inclusion and exclusion criteria for being in the study prespecified and applied uniformly to all participants?	Cant tel	NA	Yes	Yes	Yes	Cant tell	Yes	Cant tell	Yes	Yes	Yes	Yes
Was a sample size justification, power description, or variance and effect estimates provided?	No	NA	No	Yes	Yes	No	No	No	Yes	Yes	Yes	Yes
For the analyses in this paper, were the exposure(s) of interest measured prior to the outcome(s) being measured?	NA	NA	NA	No	No	NA	NA	No	Yes	NA	NA	No
Was the timeframe sufficient so that one could reasonably expect to see an association between exposure and outcome if it existed?	NA	NA	NA	No	No	NA	NA	No	Yes	NA	NA	No
For exposures that can vary in amount or level, did the study examine different levels of the exposure as related to the outcome (e.g., categories of exposure, or exposure measured as continuous variable)?	NA	NA	NA	NA	No	NA	NA	NA	Yes	NA	NA	Yes
Were the exposure measures (independent variables) clearly defined, valid, reliable, and implemented consistently across all study participants?	NA	NA	No	NA	Yes	NA	NA	NA	Yes	NA	NA	Yes
Was the exposure(s) assessed more than once over time?	NA	NA	No	No	Yes	NA	NA	NA	Yes	NA	NA	NA
Were the outcome measures (dependent variables) clearly defined, valid, reliable, and implemented consistently across all study participants?	Cant tell	Yes	No	no	Yes	Yes	Yes	Yes	Yes	Yes	Yes	Yes
Were the outcome assessors blinded to the exposure status of participants?	NA	NA	NA	NA	No	NA	No	No	No	NA	NA	No
Was loss to follow-up after baseline 20% or less?	NA	NA	NA	yes	NA	NA	NA	NA	NA	NA	NA	NA
Were key potential confounding variables measured and adjusted statistically for their impact on the relationship between exposure(s) and outcome(s)?	Yes	NA	NA	Yes	Yes	No	NA	NA	No	NA	NA	NA
Final score: over 70% (green), 50-70% (amber) and less than 50% (red)	42,86%	100,00%	37,50%	54,55%	69,23%	50,00%	57,14%	44,44%	76,92%	66,67%	66,67%	57,14%

	Hernandez 2018	Romero 2014	Rabba 2017	Padilla 2017	Fernandez 2016	Pasin 2018	Fuentes 2017	Lazou 2016	Matta 2016	Rosso 2016	Sarti 2016	Coudeville 2016
Was the research question or objective in this paper clearly stated?	Yes	Yes	Yes	Yes	Yes	Yes	Yes	Yes	Yes	Yes	Yes	Yes
Was the study population clearly specified and defined?	Yes	Yes	Cant tell	Yes	Yes	Yes	Yes	Yes	Yes	Yes	Yes	Cant tell
Was the participation rate of eligible persons at least 50%?	Cant tell	Cant tell	Yes	Yes	NA	Yes	Cant tell	Cant tell	Yes	Yes	Yes	Cant tell
Were all the subjects selected or recruited from the same or similar populations (including the same time period)? Were inclusion and exclusion criteria for being in the study prespecified and applied uniformly to all participants?	Yes	NA	NA	Yes	NA	Yes	Yes	Yes	NA	NA	No	NA
Was a sample size justification, power description, or variance and effect estimates provided?	No	NA	NA	No	NA	Yes	Yes	Yes	Yes	NA	Yes	NA
For the analyses in this paper, were the exposure(s) of interest measured prior to the outcome(s) being measured?	No	NA	NA	No	NA	Yes	NA	Yes	NA	NA	Yes	NA
Was the timeframe sufficient so that one could reasonably expect to see an association between exposure and outcome if it existed?	No	NA	NA	No	NA	Yes	NA	Yes	NA	NA	Yes	NA
For exposures that can vary in amount or level, did the study examine different levels of the exposure as related to the outcome (e.g., categories of exposure, or exposure measured as continuous variable)?	Yes	NA	NA	Yes	NA	Cant tell	NA	Yes	NA	NA	Yes	NA
Were the exposure measures (independent variables) clearly defined, valid, reliable, and implemented consistently across all study participants?	Yes	NA	NA	NA	NA	Yes	NA	Yes	NA	NA	Yes	NA
Was the exposure(s) assessed more than once over time?	NA	NA	NA	NA	NA	Yes	NA	Yes	NA	NA	Yes	NA
Were the outcome measures (dependent variables) clearly defined, valid, reliable, and implemented consistently across all study participants?	Yes	Yes	Yes	Yes	Yes	Yes	Yes	Yes	yes	Yes	Yes	NA
Were the outcome assessors blinded to the exposure status of participants?	No	NA	NA	NA	NA	No	NA	No	NA	NA	No	NA
Was loss to follow-up after baseline 20% or less?	NA	NA	NA	NA	NA	No	NA	yes	NA	NA	No	NA
Were key potential confounding variables measured and adjusted statistically for their impact on the relationship between exposure(s) and outcome(s)?	NA	NA	Yes	NA	NA	Yes	NA	Yes	NA	NA	No	Yes
Final score: over 70% (green), 50-70% (amber) and less than 50% (red)	54,55%	75,00%	80,00%	66,67%	66,67%	71,43%	83,33%	85,71%	100,00%	100,00%	71,43%	50,00%

	Castrillon 2015	Biswal 2020	Caceres 2020	Quintero 2020	Hernanadez 2020	Tarantine 2018	Marban 2020	Mercado Reyes 2019	Rosso 2019	Cardenas 2019	Carabali 2019	Piedrahita 2018
Was the research question or objective in this paper clearly stated?	Yes	Yes	Yes	Yes	Yes	Yes	Yes	Yes	Yes	Yes	Yes	Yes
Was the study population clearly specified and defined?	Yes	Yes	Cant tell	Yes	Yes	Yes	Yes	Yes	Yes	Cant tell	Yes	Yes
Was the participation rate of eligible persons at least 50%?	Cant tell	Yes	Cant tell	Yes	Cant tell	Yes	Cant tell	Cant tell	Cant tell	Cant tell	Cant tell	Yes
Were all the subjects selected or recruited from the same or similar populations (including the same time period)? Were inclusion and exclusion criteria for being in the study prespecified and applied uniformly to all participants?	Yes	Yes	NA	Yes	Yes	No	Cant tell	Yes	Yes	Yes	Yes	Yes
Was a sample size justification, power description, or variance and effect estimates provided?	No	Yes	NA	Yes	No	Yes	No	No	No	No	No	Yes
For the analyses in this paper, were the exposure(s) of interest measured prior to the outcome(s) being measured?	Yes	Yes	NA	No	Cant tell	NA	No	NO	NA	No	No	No
Was the timeframe sufficient so that one could reasonably expect to see an association between exposure and outcome if it existed?	Yes	Yes	NA	No	Yes	NA	No	No	NA	Yes	No	No
For exposures that can vary in amount or level, did the study examine different levels of the exposure as related to the outcome (e.g., categories of exposure, or exposure measured as continuous variable)?	No	Yes	NA	Yes	Cant tell	NA	NA	No	NA	Cant tell	Cant tell	Yes
Were the exposure measures (independent variables) clearly defined, valid, reliable, and implemented consistently across all study participants?	Yes	Yes	NA	Yes	Cant tell	NA	BA	NA	NA	Cant tell	Cant tell	Yes
Was the exposure(s) assessed more than once over time?	NA	Yes	NA	No	No	NA	NA	NA	NA	No	NO	No
Were the outcome measures (dependent variables) clearly defined, valid, reliable, and implemented consistently across all study participants?	Yes	Yes	NA	Yes	Yes	Yes	Yes	Yes	Yes	Yes	Yes	Yes
Were the outcome assessors blinded to the exposure status of participants?	NA	No	NA	No	No	NA	NA	NA	NA	NA	NA	No
Was loss to follow-up after baseline 20% or less?	NA	No	NA	yes	NA	NA	NA	NA	NA	No	NA	NA
Were key potential confounding variables measured and adjusted statistically for their impact on the relationship between exposure(s) and outcome(s)?	NA	Yes	NA	Yes	No	No	NA	NA	NA	Cant tell	no	Yes
Final score: over 70% (green), 50-70% (amber) and less than 50% (red)	70,00%	92,86%	33,33%	71,43%	38,46%	71,43%	37,50%	44,44%	66,67%	30,77%	33,33%	69,23%

	Castrillon 2015	Biswal 2020	Caceres 2020	Quintero 2020	Hernanadez 2020	Tarantine 2018	Marban 2020	Mercado Reyes 2019	Rosso 2019	Cardenas 2019	Carabali 2019	Piedrahita 2018	Nouvelle 2018
Was the research question or objective in this paper clearly stated?	Yes	Yes	Yes	Yes	Yes	Yes	Yes	Yes	Yes	Yes	Yes	Yes	Yes
Was the study population clearly specified and defined?	Yes	Yes	Cant tell	Yes	Yes	Yes	Yes	Yes	Yes	Cant tell	Yes	Yes	Cant tell
Was the participation rate of eligible persons at least 50%?	Cant tell	Yes	Cant tell	Yes	Cant tell	Yes	Cant tell	Cant tell	Cant tell	Cant tell	Cant tell	Yes	Cant tell
Were all the subjects selected or recruited from the same or similar populations (including the same time period)? Were inclusion and exclusion criteria for being in the study prespecified and applied uniformly to all participants?	Yes	Yes	NA	Yes	Yes	No	Cant tell	Yes	Yes	Yes	Yes	Yes	Yes
Was a sample size justification, power description, or variance and effect estimates provided?	No	Yes	NA	Yes	No	Yes	No	No	No	No	No	Yes	Cant ell
For the analyses in this paper, were the exposure(s) of interest measured prior to the outcome(s) being measured?	Yes	Yes	NA	No	Cant tell	NA	No	NO	NA	No	No	No	No
Was the timeframe sufficient so that one could reasonably expect to see an association between exposure and outcome if it existed?	Yes	Yes	NA	No	Yes	NA	No	No	NA	Yes	No	No	No
For exposures that can vary in amount or level, did the study examine different levels of the exposure as related to the outcome (e.g., categories of exposure, or exposure measured as continuous variable)?	No	Yes	NA	Yes	Cant tell	NA	NA	No	NA	Cant tell	Cant tell	Yes	NA
Were the exposure measures (independent variables) clearly defined, valid, reliable, and implemented consistently across all study participants?	Yes	Yes	NA	Yes	Cant tell	NA	BA	NA	NA	Cant tell	Cant tell	Yes	NA
Was the exposure(s) assessed more than once over time?	NA	Yes	NA	No	No	NA	NA	NA	NA	No	NO	No	NA
Were the outcome measures (dependent variables) clearly defined, valid, reliable, and implemented consistently across all study participants?	Yes	Yes	NA	Yes	Yes	Yes	Yes	Yes	Yes	Yes	Yes	Yes	Yes
Were the outcome assessors blinded to the exposure status of participants?	NA	No	NA	No	No	NA	NA	NA	NA	NA	NA	No	No
Was loss to follow-up after baseline 20% or less?	NA	No	NA	yes	NA	NA	NA	NA	NA	No	NA	NA	NA
Were key potential confounding variables measured and adjusted statistically for their impact on the relationship between exposure(s) and outcome(s)?	NA	Yes	NA	Yes	No	No	NA	NA	NA	Cant tell	no	Yes	Cant tell
Final score: over 70% (green), 50-70% (amber) and less than 50% (red)	70,00%	92,86%	33,33%	71,43%	38,46%	71,43%	37,50%	44,44%	66,67%	30,77%	33,33%	69,23%	30,00%

	Kistosik 2018	Sanchez 2017	Rodriguez 2016	Burgos 2015	Cardenas 2015	Pinzon 2015	Camacho 2014	Lim 2012	Hernandez 2016	Moreno 2016	Rojas 2020	Triana 2019	Vasquez 2020
Was the research question or objective in this paper clearly stated?	Yes	Yes	Yes	Yes	Yes	Yes	Yes	Yes	Yes	Yes	Yes	Yes	Yes
Was the study population clearly specified and defined?	Cant tell	Yes	Yes	Cant tell	Cant tell	Yes	Cant tell	Cant tell	Yes	Yes	Yes	Yes	Yes
Was the participation rate of eligible persons at least 50%?	Cant tell	Cant tell	Cant tell	Cant tell	Cant tell	Cant tell	Cant tell	Cant tell	Yes	Yes	Yes	Yes	Yes
Were all the subjects selected or recruited from the same or similar populations (including the same time period)? Were inclusion and exclusion criteria for being in the study prespecified and applied uniformly to all participants?	Yes	Yes	Yes	Cant tell	Yes	Yes	Yes	Yes	Yes	Yes	Yes	Yes	Yes
Was a sample size justification, power description, or variance and effect estimates provided?	Cant ell	Yes	No	Cant tell	Cant tell	Cant tell	Cant tell	Cant tell	Yes	No	Yes	Yes	Yes
For the analyses in this paper, were the exposure(s) of interest measured prior to the outcome(s) being measured?	No	NA	No	Cant tell	No	Cant tell	No	NA	No	NA	No	NA	NA
Was the timeframe sufficient so that one could reasonably expect to see an association between exposure and outcome if it existed?	No	NA	No	NA	No	Cant tell	No	NA	No	NA	No	NA	NA
For exposures that can vary in amount or level, did the study examine different levels of the exposure as related to the outcome (e.g., categories of exposure, or exposure measured as continuous variable)?	NA	NA	Yes	Cant tell	Yes	Yes	Yes	NA	NA	NA	NA	NA	NA
Were the exposure measures (independent variables) clearly defined, valid, reliable, and implemented consistently across all study participants?	NA	NA	Yes	NA	NA	Yes	NA	NA	NA	NA	NA	NA	NA
Was the exposure(s) assessed more than once over time?	NA	NA	No	NA	NA	No	NA	NA	NA	NA	NA	NA	NA
Were the outcome measures (dependent variables) clearly defined, valid, reliable, and implemented consistently across all study participants?	Yes	NA	Yes	Yes	NA	Yes	NA	NA	NA	NA	Yes	NA	NA
Were the outcome assessors blinded to the exposure status of participants?	No	NA	No	NA	NA	No	NA	NA	NA	NA	NA	NA	NA
Was loss to follow-up after baseline 20% or less?	NA	NA	NA	NA	NA	No	NA	NA	NA	NA	NA	NA	NA
Were key potential confounding variables measured and adjusted statistically for their impact on the relationship between exposure(s) and outcome(s)?	Cant tell	Yes	No	Cant tell	Cant tell	Cant tell	Cant tell	NA	NA	NA	Yes	Yes	NA
Final score: over 70% (green), 50-70% (amber) and less than 50% (red)	30,00%	66,67%	46,15%	22,22%	33,33%	42,86%	33,33%	40,00%	71,43%	60,00%	77,78%	100,00%	100,00%

	Salvo 2019	Castro 2016	Bracho 2017	Sheppard 2016	Zeng 2018	Molinero 2020	Tuesca 2018	Sanchez 2017	Montoya 2017	Krystosik 2016	Caribali 2015	RicoMendoza 2019	Garcia 2016
Was the research question or objective in this paper clearly stated?	Yes	Yes	Yes	Yes	Yes	Yes	Yes	Yes	Yes	Yes	Yes	Yes	Yes
Was the study population clearly specified and defined?	Yes	Yes	Yes	Yes	Yes	Yes	Yes	Yes	Yes	Yes	Yes	Yes	Yes
Was the participation rate of eligible persons at least 50%?	Cant tell	NA	Yes	Yes	Yes	Yes	Yes	Yes	Yes	Yes	Cant tell	Yes	Yes
Were all the subjects selected or recruited from the same or similar populations (including the same time period)? Were inclusion and exclusion criteria for being in the study prespecified and applied uniformly to all participants?	Yes	NA	Yes	NA	Yes	Yes	Yes	Yes	Yes	Yes	Yes	Yes	Yes
Was a sample size justification, power description, or variance and effect estimates provided?	Yes	NA	Yes	NA	Yes	Yes	Yes	Yes	Cant tell	Yes	Cant tell	Yes	Yes
For the analyses in this paper, were the exposure(s) of interest measured prior to the outcome(s) being measured?	NA	NA	No	NA	NA	No	No	No	No	No	No	No	No
Was the timeframe sufficient so that one could reasonably expect to see an association between exposure and outcome if it existed?	NA	NA	No	NA	NA	No	No	No	No	No	No	No	No
For exposures that can vary in amount or level, did the study examine different levels of the exposure as related to the outcome (e.g., categories of exposure, or exposure measured as continuous variable)?	NA	NA	Yes	NA	NA	No	NA	NA	No	NA	NA	Yes	NA
Were the exposure measures (independent variables) clearly defined, valid, reliable, and implemented consistently across all study participants?	NA	NA	Yes	NA	NA	NA	NA	NA	NA	NA	NA	Yes	NA
Was the exposure(s) assessed more than once over time?	NA	NA	NA	NA	NA	NA	NA	NA	NA	NA	NA	No	NA
Were the outcome measures (dependent variables) clearly defined, valid, reliable, and implemented consistently across all study participants?	NA	NA	Yes	NA	NA	NA	NA	NA	NA	NA	NA	Yes	NA
Were the outcome assessors blinded to the exposure status of participants?	NA	NA	No	NA	NA	NA	NA	NA	NA	NA	NA	No	NA
Was loss to follow-up after baseline 20% or less?	NA	NA	NA	NA	NA	NA	NA	NA	NA	NA	NA	No	NA
Were key potential confounding variables measured and adjusted statistically for their impact on the relationship between exposure(s) and outcome(s)?	NA	Yes	Yes	Yes	Yes	na	No	No	NA	NA	NA	Yes	Yes
Final score: over 70% (green), 50-70% (amber) and less than 50% (red)	80,00%	100,00%	75,00%	75,00%	100,00%	62,50%	62,50%	62,50%	62,50%	71,43%	42,86%	64,29%	75,00%

4. Dengue Surveillance System

In Colombia, dengue is a pathology subject to surveillance and epidemiological control, so its notification is mandatory through the National Public Health Surveillance System – SIVIGILA.

According to the Dengue surveillance protocol of the National Institute of Health (1), cases are defined as follows:

Probable case

Probable case of dengue: Patient from an endemic area who meets the definition of dengue with or without warning signs.

- **Dengue without warning signs:** Acute febrile illness of 2 to 7 days of evolution with two or more of the following manifestations: headache, retro-ocular pain, myalgia, arthralgia, skin rash or leukopenia.
- **Dengue with warning signs:** A patient who meets the previous definition and also presents any of the following warning signs: severe and continuous abdominal pain or tenderness, persistent vomiting, diarrhoea, fluid accumulation (ascites, pleural effusion, pericardial effusion), bleeding in the mucous membranes, lethargy or irritability (mainly in children), postural hypotension, painful hepatomegaly >2 cm, drop in temperature, abrupt drop in platelets (<100,000) associated with hemoconcentration.

Probable case of severe dengue: Any case of dengue that meets any of the manifestations mentioned below:

- Severe plasma extravasation: Leading to dengue shock syndrome or fluid accumulation with respiratory distress.
- Severe haemorrhages: Patient with acute febrile illness who presents severe haemorrhages with hemodynamic compromise.
- Severe organ damage: Patient with acute febrile illness and presenting clinical signs or paraclinical signs of severe organ damage such as liver damage, central nervous system damage, heart or affection of other organs.

Probable case of death due to dengue: Any case that dies with a diagnosis of severe dengue.

Laboratory confirmed case

A probable case of dengue, severe dengue, or mortality due to dengue confirmed by any of the laboratory tests for the diagnosis of dengue: ELISA NS1, RT-PCR or viral isolation

in patients with five days or less of symptom onset or Dengue IgM ELISA test in patients with six or more days of onset of symptoms. Immunochromatographic tests (rapid tests) can be used to confirm the diagnosis of dengue except in cases of probable mortality due to dengue. However, it is necessary to consider that the negative results of immunochromatographic tests for detecting NS1 and IgM do not exclude dengue infection. In this situation, additional tests should be performed to rule out the diagnosis according to the disease phase, such as RT-PCR, NS1 ELISA or IgM ELISA.

Epidemiologically linked confirmed case

The epidemiological link confirms probable dengue cases related to laboratory-confirmed cases using the association of person, time and space. With the serological information of the department, district or municipality, the epidemiological link will be used to confirm all probable cases within a perimeter of 200 meters (approximately two blocks) of another laboratory-confirmed case in the 21 days (3 weeks) before or after laboratory diagnosis.

It must be verified that when confirming probable cases, they have an epidemiological link from 21 days before and 21 days after confirming a case by the laboratory in the same neighbourhood.

Mortality for dengue

Dengue mortality is any death of a severe dengue case with a confirmed diagnosis based on clinical characteristics, epidemiological link or laboratory tests (serum sample for IgM ELISA, NS1 ELISA, viral isolation or RT-PCR in serum or tissues or compatible histopathology).

Any probable case that dies with a clinical diagnosis of severe dengue without adequate tissue or serum sampling to perform virological tests will be considered a compatible case of death from dengue and represents a failure of the epidemiological surveillance system.

References

1. Colombia, Instituto Nacional de Salud. Protocolo de Vigilancia del Dengue. Versión 4. 2022.

5. Number of dengue cases and deaths related to in Colombia from 2012 to 2020.

Year	Incidence				Number of deaths	
	Dengue cases		Severe dengue cases		Probable n	Confirmed n
	Probable n (%)	Confirmed n (%)	Probable n (%)	Confirmed n (%)		
**2012**	25774 (49.2%)	26693 (50.8%)	501 (35%)	911 (65%)	17	71
**2013**	60 309 (49%)	62 132 (51%)	838 (27%)	2 275 (73%)	35	148
**2014**	53.745 (51%)	51.611 (49%)	736 (28,8%)	1.883 (71,2%)	48	118
**2015**	56697 (59,7%)	38326 (40,3%)	327 (23%)	1094 (77%)	54	101
**2016**	52164 (52,1%)	47953 (47,9%)	140 (15,6%)	759 (84,4%)	42	84
**2017**	17343 (69,2%)	7705 (30,8%)	74 (31,4%)	162 (68,6%)	7	15
**2018**	20555 (47,1%)	23097 (52,9%)	144 (27,7%)	375 (72,3%)	27	40
**2019**	49582 (40,1%)	74059 (59,9%)	172 (12,8%)	1176 (87,2%)	24	98
**2020**	33856 (44,3%)	42563 (55,7%)	154 (17,9%)	708 (82,1%)	19	51

6. National dengue serotype distribution, 2012-2015.

Year	Total samples	DENV-1		DENV-2		DENV-3		DENV-4	
	n	n	%	n	%	n	%	n	%
**2012**	326	179	55%	49	15%	78	24%	20	6%
**2013**	763	412	54%	92	12%	214	28%	46	6%
**2014**	827	248	30%	240	29%	223	27%	116	14%
**2015**	196	14	7%	131	67%	46	24%	5	3%
